# The role of extracorporeal membrane oxygenation in critically ill patients with COVID-19: a narrative review

**DOI:** 10.1186/s12890-021-01479-6

**Published:** 2021-04-08

**Authors:** Shiqian Huang, Shuai Zhao, Huilin Luo, Zhouyang Wu, Jing Wu, Haifa Xia, Xiangdong Chen

**Affiliations:** 1 Department of Anaesthesiology, Union Hospital, Tongji Medical College, Huazhong University of Science and Technology, No. 1277, Jiefang Avenue, Wuhan, 430022 China; 2 Department of Anaesthesiology, Wuhan Red Cross Hospital, Wuhan, 430015 China

**Keywords:** COVID-19, SARS-CoV-2, ECMO, ARDS, COVID-19- and ECMO-related pathophysiology, Recommendations

## Abstract

Extracorporeal life support treatments such as extracorporeal membrane oxygenation (ECMO) have been recommended for the treatment of severe acute respiratory distress syndrome (ARDS) patients with coronavirus disease 2019 (COVID-19). To date, many countries, including China, have adopted ECMO as a treatment for severe COVID-19. However, marked differences in patient survival rates have been reported, and the underlying reasons are unclear. This study aimed to summarize the experience of using ECMO to treat severe COVID-19 and provide suggestions for improving ECMO management. The effects of severe acute respiratory syndrome coronavirus 2 (SARS-CoV-2) on the pathophysiology of COVID-19 and the effects of ECMO on the clinical outcomes in patients with severe cases of COVID-19 were reviewed. Recent data from frontline workers involved in the use of ECMO in Wuhan, China, and those experienced in the implementation of artificial heart and lung support strategies were analysed. There is evidence that ECMO may complicate the pathophysiological state in COVID-19 patients. However, many studies have shown that the appropriate application of ECMO improves the prognosis of such patients. To expand our understanding of the benefits of ECMO for critically ill patients with COVID-19, further prospective, multicentre clinical trials are needed.

## Background

The current outbreak of coronavirus disease 2019 (COVID-19), which is caused by severe acute respiratory syndrome coronavirus 2 (SARS-CoV-2), has affected millions of people worldwide [1]. As of January 22, 2021, more than 2 million deaths due to COVID-19 had been recorded worldwide, according to the latest report from Johns Hopkins University and other sources [2]. Approximately 15% to 30% of people infected by SARS-CoV-2 develop acute respiratory distress syndrome (ARDS) and are at a high risk of mortality [3]. Despite meeting most of the criteria in the Berlin definition of ARDS, COVID-19 has unique pathophysiological characteristics, such as progressive hypoxic dyspnoea, inflammatory cytokine storms and hypercoagulability [4–7]. In general, there are two primary phenotypes of hypoxemic respiratory failure: Type L (“non-ARDS”), which is characterized by normal or high compliance and low recruitability, and Type H (“typical ARDS”), which is characterized by very low compliance and high recruitability, together with severe hypoxia [8].

ECMO is initiated in cases of refractory hypoxemia that are unresponsive to conventional care. Veno-venous (V-V) ECMO is mainly used in patients with severe COVID-19-related ARDS, and a small number of patients with circulatory disorders are managed with veno-arterial (V-A) or veno-venous arterial (V-VA) ECMO. The prognosis is often worse in the latter groups of patients. It is worth noting that ECMO can also affect normal physiology and exacerbate coagulation and immune abnormalities in patients with COVID-19 despite having good therapeutic effects (Table 1) [4, 9]. The outcomes in COVID-19 patients after receiving ECMO treatment have varied substantially in different studies, probably due to differences in clinical, organizational and resource management factors [10–16]. Recommendations from the World Health Organization (WHO) published in August 2020 stated that the use of ECMO for the treatment of severe ARDS due to COVID-19 should be offered only in expert centres with sufficient experience [17]. As more data accumulate, there is a need to discuss the role of ECMO in patients with COVID-19. In this review, we summarize the pathophysiological characteristics of COVID-19 patients and the effects and clinical outcomes of treatment with ECMO. Furthermore, we provide recommendations for the use of ECMO [18–22].

**Table 1:** The influence of the SARS-CoV-2 on the pathophysiology of severe COVID-19 patients and ECMO-related therapeutic effects and complications

	SARS-CoV-2	ECMO
Respiratory system	Patients with COVID-19 are mainly predominant by respiratory failure. The pathophysiology of progression to ARDS in COVID-19 patients is very complicated. SARS-CoV-2 directly attacks enough alveoli epithelial cells via the ACE2 receptor to cause pulmonary edema, hyaline membrane formation and collapse of lobular of the lungs. Hypoxic pulmonary vasoconstriction failure, pulmonary embolism and/or pulmonary microcirculation embolism, abnormal immune response may also contribute to the development of ARDS	V-V ECMO mainly provides therapeutic benefits to the respiratory system by improving oxygenation and promoting lung-protective ventilation
Cardiovascular system	It is the second cause of death after respiratory failure in COVID-19 patients. There are several possible mechanisms contributing to cardiac injury: direct injury from viral toxicity; oxygen supply-to-demand mismatch cause damage to myocardial cells; abnormal coagulation, microvascular dysfunction and plaque rupture; systemic inflammatory response and immune system disorders bringing stress to the failing myocardium and leading to further depression in myocardial function	V-A ECMO can support highly selected cases with clear evidence of refractory left ventricle dysfunction. When differential hypoxemia complicating V-A ECMO happens, hybrid V-V/V-A ECMO can be used as a remedial option. A small number of patients using ECMO could bring cardiovascular complications, including atrial thrombosis, fatal arrhythmia, etc
Blood system	*Coagulation*: COVID-19 patients are characterized by a hypercoagulable state, and associated with a high incidence of thrombosis. The possible mechanism is that the direct impact of SARS-CoV-2 or related excessive inflammatory state leads to the activation of blood coagulation function *Immune response*: Higher-risk COVID-19 subgroups tended to have lymphopenia yet with overall leukocytosis and high inflammatory markers (CRP, fibrinogen, ferritin, IL-6). The SARS-CoV-2 attack of cytoplasmic component of the lymphocyte, spleen and lymph nodes, and the intensification in local and systemic inflammation may contribute to lymphopenia	*Coagulation*: ECMO can cause abnormal blood coagulation function, which can lead to thrombosis and bleeding events. Thrombosis events in COVID-19 patients receiving ECMO treatment is more common *Immune response*: On the one hand, ECMO technology have a certain effect of inflammatory activation. On the other hand, ECMO can reduce systemic inflammation indicators by protecting lung ventilation and completely reversing the state of systemic hypoxia It is worth noting that adequate level of anticoagulant therapy with unfractionated heparin during ECMO is a very huge challenge clinically, because of the COVID-19-related prothrombotic state and the high risk of HIT trigger
Cerebrovascular	The incidence of ischemic stroke in patients with COVID-19 is 10.3%, and most patients have conventional stroke risk factors. Hemorrhagic stroke is relatively rare, with an incidence of 0.9%. The coagulation abnormalities of COVID-19 contributed to this result	The most common ECMO-related cerebrovascular complication is cerebral hemorrhage, with rates up to 21%
Liver	Hepatic dysfunction was seen in 14–53% of COVID-19 patients, particularly in patients hospitalized in ICU (62%), while severe liver failure is rare. SARS-CoV-2 involvement may be related to viral direct damage to liver cells, severe infection, uncontrolled immune response	ECMO is not recommended for patients with severe liver failure
Kidney	The prevalence of AKI among COVID-19 patients ranges from 0.5% to 5.1%, and its severity were highly correlated with poor outcomes. The potential mechanisms may include three aspects: cytokine damage, organ crosstalk and systemic effects	Improvement in kidney oxygenation due to V-V ECMO could be favorable to kidney recovery. Though, ECMO may also aggravate kidney damage by promoting cytokine generation

*SARS-CoV-2* severe acute respiratory syndrome coronavirus 2, *ECMO* extracorporeal membrane oxygenation, *COVID-19* Coronavirus disease 2019, *ARDS* acute respiratory distress syndrome, *ACE2* angiotensin-converting enzyme 2, *CRP* C-reactive protein, *ICU* intensive care unit, *V-V* veno-venous, *V-A* veno-arterial, *HIT* heparin-induced thrombocytopenia, *AKI* acute kidney injury

## Pathophysiological characteristics of critically ill patients with COVID-19

Scientists have determined that SARS-CoV-2 is a β-coronavirus, a group that includes MERS CoV, SARS-CoV-1, and HCoV-OC43 [23]. SARS-CoV-2 enters the epithelial cells of the respiratory and gastrointestinal tracts with the help of the angiotensin-converting enzyme 2 (ACE2) receptor. The risk factors that contribute to death in patients with severe COVID-19 are ARDS severity; older age; comorbidities, such as hypertension, obesity and diabetes; secondary infections; and elevated inflammatory markers in the blood [5, 13–16].

### Respiratory system

In intensive care unit (ICU) patients with COVID-19, acute hypoxemic respiratory failure of varying severity is a common feature, and its incidence can be as high as 67% [15]. Preliminary clinical studies have confirmed that the mortality rate in critically ill patients is 50–90%, and hypoxic respiratory failure is the main cause of death [15, 24–26]. The progression of ARDS in COVID-19 patients is very complicated, and most studies indicate that SARS-CoV-2 directly attacks enough alveolar epithelial cells to cause pulmonary oedema, hyaline membrane formation and collapse of the lobes of the lungs [27, 28]. Endothelial injury may also lead to hypoxic pulmonary vasoconstriction failure, thereby affecting pulmonary vascular function and leading to ventilation-perfusion mismatch. In addition, lung vascular thrombosis and/or pulmonary embolism may aggravate hypoxemia [29]. High levels of secreted cytokines secondary to the activation of neutrophils may also contribute to the development of ARDS [30].

There are two primary phenotypes of hypoxemic respiratory failure: Type L (“non-ARDS”), which is characterized by normal or high compliance, a low ventilation-to-perfusion ratio, low lung weight and low recruitability, and Type H (“typical ARDS”), which is characterized by very low compliance, high lung weight and high recruitability, accompanied by severe hypoxia [8, 24, 31]. The evolution from the L to the H phenotype, i.e., from a clinical state of low elastance and low lung weight to classic ARDS, may be due to the progression of COVID-19 and lung injury caused by high-stress ventilation. It is crucial to avoid high inspiratory pressure in the early stage of acute respiratory failure in COVID-19 (L phenotype). Early intubation is critical for controlling drive pressure in the L phenotype, and the use of ECMO should be limited to those with the H phenotype.

### Cardiovascular system

In addition to pulmonary complications such as ARDS, SARS-CoV-2 infection can also cause cardiovascular damage, making it the second leading cause of death in COVID-19 patients. The reported incidence of acute myocardial injury has varied from 7 to 28% among hospitalized patients, and high levels of cardiac troponin are associated with more severe disease [26, 32, 33]. Ventricular tachycardia and fibrillation are late manifestations of COVID-19 that are associated with a higher risk of mortality, and their incidence is significantly higher in patients with signs of myocardial damage. Although they have been reported in several studies, the true prevalences of COVID-19-related acute myocarditis and acute coronary syndrome remain unclear [34–36]. In addition, < 10% of patients have obvious evidence of shock, which is more common in hospitalized ICU patients [32]. Several mechanisms may contribute to cardiac injury, such as direct injury, oxygen supply-to-demand mismatch, abnormal coagulation, microvascular dysfunction, plaque rupture, the systemic inflammatory response and immune system disorders [37, 38].

### Blood coagulation and immune system

COVID-19 patients present with a hypercoagulable state, which is more common and more pronounced in ICU patients. The prevalence of a hypercoagulable state exceeds 30% in all ICU patients, whereas that of pulmonary embolism is 16.7% [4, 39]. Elevated D-dimer and fibrinogen levels are risk factors for ARDS and death in patients with COVID-19 [15, 30]. The features of COVID-19 coagulopathy include a high number of thrombotic accidents without myocardial infarction and a thrombotic state without disseminated intravascular coagulation (DIC) [4]. Thrombosis in COVID-19 patients is caused directly by SARS-CoV-2 or due to an excessive inflammatory state that activates blood coagulation.

COVID-19 patients often present with immune dysfunction and abnormal inflammation activation. Higher-risk subgroups of COVID-19 patients tend to have lymphopenia accompanied by overall leucocytosis and high levels of inflammatory markers (C-reactive protein [CRP], fibrinogen, ferritin, IL-6) [40]. High IL-6 levels and lymphopenia have been proposed as predictors of disease progression [41].

### Other organs

The incidence of ischaemic stroke in patients with COVID-19 is 10.3%, and most patients have conventional stroke risk factors. Haemorrhagic stroke is relatively rare, with an incidence of approximately 0.9% [42–44]. The coagulation abnormalities caused by infection with SARS-CoV-2 contribute to the incidence of haemorrhagic stroke. The incidence of acute kidney injury (AKI) in COVID-19 patients ranges from 0.5 to 5.1%, affecting nearly 29% of critically ill patients with COVID-19 who need ECMO support. Severe AKI is associated with poor outcomes in COVID-19 patients [30, 45, 46]. Hepatic dysfunction has been observed in 14%–53% of COVID-19 patients, particularly in patients hospitalized in the ICU (62%) [47]. Although patients with severe COVID-19 have a higher incidence of liver dysfunction, cases of severe liver failure are rare.

## ECMO and COVID-19

### V-V ECMO and V-A ECMO in COVID-19 patients

ECMO is a form of extracorporeal life support (ECLS) that is mainly used to oxygenate the blood to temporarily compensate for failing lungs or a failing heart while minimizing further iatrogenic ventilator-induced lung injury (VILI). ECMO operates mainly under two modes, V-V and V-A, depending on the auxiliary organ. In addition, variants of the above modes, such as the V-AV ECMO mode, are available. V-V ECMO is used in patients with respiratory failure only, whereas V-A ECMO is used in patients with both respiratory and circulatory failure.

Patients with COVID-19 present with respiratory failure, and the ECMO mode mainly used in these patients is V-V (91%) [48]. However, SARS-CoV-2 can also attack the cardiovascular system, resulting in circulatory failure, and the V-A ECMO mode is used in this situation. The reported rate of the use of cardiorespiratory combined ECMO support (V-A or V-AV ECMO) among COVID-19 patients is less than 10%. Notably, patients receiving V-A or V-AV ECMO have been found to have a poor prognosis. A recent multicentre study from the Extracorporeal Life Support Organization (ELSO) registry reported that the use of ECMO for circulatory support is associated with higher in-hospital mortality (hazard ratio 1.89, 95% CI 1.20–2.97) [16]. The risk factors for a poor outcome for patients undergoing ECMO during the COVID-19 pandemic are old age, low PaO_2_/FiO_2_, immunocompromised status, comorbidities, and the need for V-A ECMO.

### ECMO-related complications in COVID-19 patients

The most common complications of ECMO in COVID-19 patients include bleeding and thrombosis. In addition to the promotion of thrombosis by COVID‐19, the use of ECMO can also affect blood coagulation function. The incidence of venous thrombosis in COVID-19 patients receiving ECMO is 33%, the incidence of pulmonary embolism is 29%, and the incidence of cerebral haemorrhage is 5%-6%, whereas severe bleeding in these patients is relatively rare [4, 39, 49]. Notably, the initial establishment of ECMO results in an overall procoagulant effect. As the time goes, coagulation factors irreversibly bind with the ECMO surface coating material; therefore, they are eliminated. ECMO technology also modulates inflammatory activation. Previous studies reported that ECMO reduces systemic inflammation indicators to varying degrees by protecting lung ventilation and completely reverses the state of systemic hypoxia. Therefore, in COVID-19 patients, the benefit of the early use of ECMO to reduce systemic hypoxia exceeds the risks associated with the systemic inflammatory response caused by ECMO use [50].

Mechanical-related complications, including pump failure, oxygenator dysfunction, and circuit embolism, have also been reported in COVID-19 patients. Circuit changes, oxygenator failures, pump failures or cannula conditions occur in 28% of patients, as reported by the ELSO Registry of ECMO in COVID-19 [16], compared with an oxygenator replacement rate of 6.6% in non-COVID-19 patients [51].

### Clinical outcome in COVID-19 patients receiving ECMO

Recent studies reported that ECMO can be successfully used in appropriately selected COVID-19 patients with severe ARDS [24, 52–55]. However, the survival rate for COVID-19 patients with cardiorespiratory failure undergoing ECMO varies significantly in different countries and medical centres (Table 2).

**Table 2:** Reported clinical characteristics and ECMO outcomes for severely COVID-19 patients

Study	Spatiotemporal information	Total number of patients	ECMO Usage	Duration of ECMO use (days)	ICU overall morality, n (%)	ECMO outcome	ECMO morality, n (%)
Yang X [10]	21 ICUs in Hubei, China from late December, 2019, to May 31, 2020	73 ICU patients	Usage rate 100% (73/73), all V-V ECMO	18.5 (12–30)	59 (80.8%)	10 discharged from ICU,2 successfully weaned off ECMO but remain on mechanical ventilation, 2 remains on ECMO, 59 died	59(80.8%) for 60-days
Zhang G [56]	In Zhongnan Hospital of Wuhan University (Wuhan, China) from January 2, 2020 to February 10, 2020	44 ICU patients	Usage rate 23% (10/44), unknown mode	Unknown	9 (21%)	2 discharged, 5 remains on ECMO, 3 died	3(30%)
Zeng Y [12]	Two medical centers of Wuhan, China, early stage of COVID-19	12 ICU patients	Usage rate 100% (12/12), unknown mode	11.3 (3–28)	5 (42%)	3 improved without ECMO, 4 still alive on ECMO but 2 with coma, other 5 died	5(42%)
Li X [57]	In Shanghai, China from January 30, 2020 to March 25, 2020	8 ICU patients	Usage rate 100% (8/8), 7 V-V ECMO, 1 V-A ECMO	37 (18–47), expect V-A ECMO (3 h)	4 (50%; 3 V-V ECMO, 1 V-A ECMO)	4 died, 3 successfully weaned off ECMO but remain on mechanical ventilation, 1 still on V-V ECMO with mechanical ventilation	4(50%)
Belhadjer [53]	Twelve hospitals in France and one hospital in Switzerland from March 22, 2020 to April 30, 2020	35 child patients with febrile cardiogenic shock or left ventricular dysfunction and inflammatory state	Usage rate 28% (10/35), all V-A ECMO	4.5 (3–6)	0 (0%)	ECMO was successfully weaned in all	0 (0%)
Osho [25]	First month of the COVID-19 outbreak in Massachusetts	6 ICU patients	100% (6/6), all V-V ECMO	12 (4–18)	1 (17%)	1 died on ECMO, 5 alive with 4 successfully decannulated, including 2 successfully extubated and 1 discharged	1 (17%)
Jacobs [54]	In USA from March 17, 2020 to April 9, 2020	32 ICU patients	Usage rate 100% (32/32), 27 on V-V ECMO, 5 on V-A ECMO	6 (5–10)	10 (31%)	10 died before or shortly after decannulation, 22 alive (17 remain on ECMO, 5 successfully decannulated and extubated)	10(31%)
Le Breton [55]	In Paris from March 8, 2020 to April 18, 2020	13 ICU patients	Usage rate 100% (13/13), all V-V ECMO	13 (3–34)	2 (15%)	11 alive (discharged from ICU) with 2 died on mechanical ventilation	2 (15%)
Kon [58]	In New York University Langone Health (NYULH) Manhattan campus from March 10, 2020 to April 24, 2020	27 ICU patients	Usage rate 100% (27/27), all V-V ECMO	11 (10–14)	1 (4%)	26 alive (13 remain on ECMO, 13 successfully decannulated) with 1 died on ECMO	1 (4%)
Schmidt M [11]	In Paris–Sorbonne University Hospital from March 8, 2020 to May 2, 2020	492 ICU patients	Usage rate 16.9% (83/492), 81 (97%) on V-V ECMO, 1 (1%) on V-A ECMO, 1 (1%) on V-VA ECMO	20 (10–40)	Unknown	48 alive and discharged from the ICU, 5 alive and still in the ICU, 30 died	30(36.1%)
Barbaro RP [16]	At 213 hospitals in 36 countries from Jan 16, 2020 to May 1, 2020	1035 patients aged 16 years or older	Usage rate 100% (1035/1035), 978 (94%) on V-V ECMO, 44 (4%) on V-A ECMO, 9 (0.9%) on V-VA ECMO, and 4(0.4%) received other ECMO support	13.9(7.8–23.3)	Unknown	968 (94%) of 1035 patients were discharged from hospital alive (588), died (380)	380 (39%)

*ECMO* extracorporeal membrane oxygenation, *COVID-19* Coronavirus disease 2019, *ICU* intensive care unit, *V-V* veno-venous, *V-A* veno-arterial, *V-VA* veno-venous arterial

Previous clinical studies from China reported that patients who underwent ECMO had poor outcomes, with mortality ranging from 30 to 83% [12, 15, 56, 57]. A retrospective case series from 2 ICUs in Hubei, China enrolled 12 ECMO-treated COVID-19 patients with a mean follow-up period of 11.3 days and reported a morality rate of 41.7% [12]. Yang et al. performed a study with 60-day long-term follow-up of 73 COVID-19 patients treated with ECMO in 21 ICUs in Hubei, China, and reported a mortality rate of 80.8% [10]. The patients’ median age was 62 (range 33–78) years, whereas 23 (31.5%) were aged ≥ 65 years. The median PaO_2_/FiO_2_ ratio was 71.9 (IQR 58.6–87.0) before ECMO initiation, and 58.9% of the patients underwent prone positioning. Old age (> 65 years) has been identified as a risk factor for mortality in critically ill COVID-19 patients.

However, a study on the use of V-V ECMO in COVID-19 patients (n = 32) carried out in Japan reported a 67% ECMO weaning rate, with 2% mortality, with the remaining patients still supported [52]. Early outcomes of ECMO support in COVID-19 patients in a single institution in the United States showed an overall survival rate of 96%, and 48% of the patients were successfully decannulated [58]. Moreover, the use of V-A ECMO for multisystem inflammatory syndrome in children (MIS-C)-related cardio-circulatory impairment was found to be associated with a 100% survival rate [53]. Previous studies reported a relatively better prognosis in children and a rapid resolution of systolic dysfunction due to differences in the mechanism of COVID-19-related cardiogenic shock in children and adults. In children, myocardial involvement is due to stunning or oedema rather than inflammatory damage, and rapid recovery with the use of immunoglobulin and steroids has been reported. Schmidt et al. explored data from 83 COVID-19-related ARDS patients (median age 49 [IQR 41–56] years; 61 [73%] men) in the Paris–Sorbonne University Hospital Network, which is composed of 5 ICUs [11]. Patients presented with severe ARDS with a median PO_2_/FiO_2_ of 60 (IQR 54–68) mmHg before ECMO initiation. Further analysis showed that the estimated 60-day mortality rate was 31% (95% CI 22–42), which is similar to the rate reported in the EOLIA trial and the large prospective LIFEGARD registry of ECMO for severe ARDS not caused by COVID-19 [59]. Notably, 78 (94%) patients received adjuvant therapy, including prone positioning before ECMO initiation and a homogeneous ultraprotective ventilation strategy with tight control of the driving pressure during ECMO. In October 2020, the ELSO registry published the largest multicentre study to date, involving 1035 COVID-19-related ECMO patients from 213 centres across 36 countries. This multicentre study reported a mortality rate of 37% (95% CI 34–40) after ECMO initiation [16]. The median age of the patients included in the study was 49 years (IQR 41–57), and 724 (70%) patients had at least one pre-ECMO comorbidity. The median PaO_2_/FiO_2_ ratio prior to ECMO use was 72 (IQR 59–94), and 60% of the patients underwent prone positioning prior to ECMO initiation. The risk factors identified in this study were age, immunocompromised state, chronic respiratory disease, pre-ECMO cardiac arrest, severe hypoxaemia, acute kidney injury, and the use of ECMO for temporary circulatory support (V-A ECMO support vs. V-V ECMO support).

This significant discrepancy in survival can be attributed to several factors, including variations in viral virulence. The Chinese scientific research team has reported that SARS-CoV-2 has accumulated approximately 149 mutations and has evolved to two subtypes, namely, the L subtype and the S subtype [60]. Different virus subtypes have different levels of virulence and infectivity. The highly virulent L subtype was more common in the early stages of the Wuhan outbreak. In addition, the difference in mortality can be attributed to the pre-pandemic intensive care infrastructure and resource constraints imposed by the pandemic. During the COVID-19 epidemic, there were insufficient medical resources available, especially in the most severely affected location, Wuhan City, the capital of Hubei Province. Studies at the time reported that 15.5% of cases in Wuhan, China, were severe and critical. The Chinese government deployed more than 40,000 medical staff members from other provinces to Hubei Province to control the epidemic [61]. The ECMO centre in the report by Schmidt and colleagues is highly experienced, and the hospital-level volume of ECMO cases may contribute to better patient outcomes after ECMO use [11]. Furthermore, different pre-ECMO management strategies are closely related to patient prognosis, including prone positioning, lung-protective ventilation, neuromuscular blockade, and inhaled nitric oxide. Schmidt et al. reported a high rate of pre-ECMO prone positioning in COVID-19 patients (94%) compared with a rate of 58.9% in the COVID-19 patients in the report by Yang et al. Patients with the most severe form of ARDS can benefit from the optimization of care. Moreover, the high mortality rate in China can be attributed to the fact that the median age of COVID-19 patients undergoing ECMO was relatively high. The median age of patients undergoing ECMO was 62 (range 33–78) years, as reported by Yang et al., compared with 49 (IQR 41–56) years reported by Schmidt M et al. and 49 years (IQR 41–57) reported by the ELSO registry [10, 11, 16]. Notably, 31.8% of the patients reported by Yang et al. were more than 65 years old. Age (> 65 years) is a risk factor for mortality in COVID-19 patients. Moreover, the patient population, pre-ECMO comorbidities, indications for ECMO treatment, choice of ECMO mode, and variability of follow-up time can all affect the efficacy of ECMO.

## Recommendations for using ECMO in COVID-19 patients

These marked differences in survival imply that there is a need to explore the various experiences with ECMO treatment and standardize ECMO treatment to obtain the maximum benefit. Analysis of the pathophysiological characteristics of COVID-19, their interactions with ECMO, the preliminary clinical outcomes and resource allocation problems that may cause ethical dilemmas show that ECMO can be used effectively in selected COVID-19 patients. The indicated population and selection of the mode of ECMO are discussed below.

### Indications for ECMO in patients with COVID-19

The selection of patient for ECMO should not deviate from the existing guidelines. However, due to limited capacity during a pandemic, young, previously healthy patients with single organ failure should be given priority for ECMO as they are likely to derive the maximum benefit (Table 3) [62, 63]. ELSO recommends that ECMO should be used as a rescue therapy after the failure of standard treatment approaches, including optimal ventilation strategies, neuromuscular blockade, appropriate positive end-expiratory pressure (PEEP) and prone positioning [22]. Previous studies reported that the early initiation of ECMO in COVID-19 patients with ARDS is beneficial, especially among younger patients [10, 16]. However, due to a shortage of personnel and equipment, the early application of ECMO may be impractical during the COVID-19 epidemic. A recent study based on a target trial of the treatment of severe hypoxemic respiratory failure in 190 patients treated with V-V ECMO and 1167 receiving conventional ventilation reported that V-V ECMO reduced mortality from 65 to 45% in selected cases (young patients, with no severe comorbidities, managed with ECMO within seven days after tracheal intubation) [64]. However, the crucial elements of a successful ECMO policy for COVID-19 patients should be considered. First, any ECMO centre should have a high level of personnel training and expertise and the relevant equipment and facilities. Second, ECMO services should be centrally coordinated by a "leading" ECMO centre. Third, ECMO should not be extensively used during a pandemic.

**Table 3:** Indications and Contraindications for ECMO use in COVID‐19 patients

	Items	Explanation
Indications	(1) Refractory ARDS despite optimal ventilation strategies (curare use, prone positioning, inhaled nitric oxide, etc.)	① Prone positioning is strongly recommended unless clear contraindications to prone positioning, as hemodynamic instability could justify ECMO employ without previous clinical trial in prone positioning ② Inhaled nitric oxide could be considered, but it is not mandatory before using ECMO
	(2) Prolonged mechanical ventilation < 7 d	Prolonged mechanical ventilation with ventilation settings (FiO_2_ > 0.9, plateau pressure > 30 cmH_2_O) could cause irreversible lungs injury and multiple organ damage
	(3) The use of ECMO should be considered when the risk of death is more than 50%, and should be started when the risk of death reaches or exceeds 80%	① Mortality risk greater than 50% is measured as PaO_2_/FiO_2_ < 150 and FiO_2_ > 90% and/or Murray score 2–3 [62, 63]; Mortality risk greater than 80% is measured as PaO_2_/FiO_2_ < 100 and FiO_2_ > 90% and/or Murray score 3–4 despite optimal care for 6 h or less ② Earlier use of ECMO after respiratory failure onset (1–2 days) is more likely to benefit patients with COVID-19
	(4) Severe air leak syndrome	
	(5) Complicated with severe myocarditis or cardiogenic shock	Cardiogenic shock is defined as CI < 1.8 L/min/m^2^ or MAP < 60 mmHg with maximum dose of vasoactive drugs (norepinephrine > 1 mcg/kg/min) or Intra-Aortic Balloon Pump
Con-indications	(1) Age ≥ 65 years (relative contraindications)	
	(2) Significant underlying comorbidities that cannot be recovered	Comorbidities include: CKD ≥ III, cirrhosis, dementia, advanced lung disease, uncontrolled diabetes with chronic end-organ dysfunction, severe peripheral vascular disease, severe brain dysfunction, severe damage to the central nervous system, and advanced malignant tumors
	(3) Severe immunosuppression	Absolute neutrophil count < 0.4 × 10^9^ /L
	(4) Contraindications to anticoagulation	Contraindications to anticoagulation include: liver failure caused by COVID-19 combined with severe coagulopathy, major bleeding, and recent or enlarged intracranial bleeding
	(5) Severe multiple organ failure	
	(6) Patients who are diagnosed with acute aortic dissection	
	(7) Inability to accept blood products	

*ECMO* extracorporeal membrane oxygenation, *COVID-19* Coronavirus disease 2019, *MAP* mean arterial pressure, *CKD* chronic kidney disease

### ECMO mode selection (Table 4)

**Table 4:** Special considerations for V-V, V-A and V-VA ECMO use in COVID‐19 [22]

	Items	Explanation
V-V ECMO	(1) Large multi-stage, drainage cannula is recommended (e.g. 23 Fr or 11 greater for adults)	It's possible to minimize the need for insertion of an additional drainage cannula at later stage
	(2) Dual lumen cannula should be avoided if possible	Dual lumen cannula is relatively difficult to insert, is associated with higher risk of thrombotic complications and malpositioning requiring repeat echocardiography
	(3) It's recommended that either the femoro-femoral or femoro-internal jugular configuration be used	The femoro-femoral approach allows for more rapid surgical field preparation, creates efficiency of movement around the bed, and keeps the operator away from the patient's airway
V-A and V-VA ECMO	(1) A femoro-femoral configuration for V-A ECMO cannulation is recommended	
	(2) A distal limb perfusion catheter is strongly recommended to reduce the risk of limb ischemia	
	(3) It's recommended to place three separate single lumen cannulas for the utilization of V-VA ECMO and not recommended to use a double lumen cannula for V-VA ECMO	
	(4) The initiation of V-VA ECMO as a pre-emptive strategy is not recommended	If a patient requires V-V ECMO but has no evidence of cardiac dysfunction or cardiac dysfunction is medically supportable with inotropes, placement of an arterial cannula is strongly discouraged

*ECMO* extracorporeal membrane oxygenation, *COVID-19* Coronavirus disease 2019, *V-V* veno-venous, *V-A* veno-arterial, *V-VA* veno-venous arterial

#### V-V ECMO

The lungs are the most vulnerable organs in COVID-19 patients, and most patients have normal cardiac function in the early stage. V-V ECMO is the primary ECMO mode used in patients with ARDS, as it provides a “time window” during which the failing lungs can rest and recover. V-V ECMO provides full or partial extracorporeal pulmonary support by adjusting the blood flow (which can be as high as 7 L/min) [65]. The oxygen flow support by ECMO needed in patients with advanced COVID-19-related respiratory distress, especially in obese patients, can be as high as 5 L/min to meet the systemic oxygen demand [25]. The contribution of ECMO to arterial oxygenation depends mainly on the patient's lung function and should be accurately adjusted based on the patient's condition. V-V ECMO only provides gas exchange and does not provide direct haemodynamic effects, unlike V-A ECMO. Perfusion of the body still depends on the pumping of the patient's own heart. An echocardiographic examination should be performed before starting the V-V ECMO circuit to confirm whether there is severe left heart insufficiency, which may require the placement of a V-A ECMO circuit [66].

V-V ECMO drains blood from a femoral venous or internal jugular venous cannula. The blood is then pumped through a membrane oxygenator, and finally, it is returned to the venous system either through a femoral venous or internal jugular venous cannula. A large multi-stage drainage cannula is recommended (such as 23 Fr or greater for adults) to minimize the need for the insertion of an additional drainage cannula at a later stage. The right femoral vein and the right internal jugular vein (which are relatively straight) are often used as the preferred vessels for V-V ECMO catheterization based on the availability of resources, personnel experience and ease of operation [52]. Improper placement can easily increase recirculation and drain oxygenated blood from the body, reducing the oxygenation efficiency of ECMO. Therefore, chest X-ray or transthoracic ultrasound should be performed after peripheral cannulation to confirm the correct position [67]. The Seldinger technique, which can be performed by nonsurgical staff and without surgical equipment, is also recommended for cannulation, as it does not require skin sutures, it reduces bleeding and reduces the risk of SARS-CoV-2 infection [68].

#### V-A ECMO

V-A ECMO is a form of ECLS that provides temporary mechanical circulatory support and simultaneous extracorporeal gas exchange in patients with severe cardiogenic shock and decompensated heart failure. The initiation of V-A ECMO serves as a salvage intervention in COVID-19 patients with cardiogenic shock or cardiac arrest. The timely initiation of V-A ECMO is recommended prior to the development of multiple organ failure. V-A ECMO drains deoxygenated blood from the right atrium through a femoral venous or internal jugular venous cannula and pumps it through a membrane oxygenator, allowing oxygenation and carbon dioxide removal. The oxygenated blood is then returned to arterial circulation through a cannula placed in a peripheral artery, usually the femoral or subclavian artery. A femoro-femoral configuration is recommended for V-A ECMO cannulation.

A common complication of V-A ECMO is hypoxia in the upper body, which can cause severe cerebral hypoxia. Hypoxia in the upper body (a lower PaO_2_ in the upper body than in the lower body), also known as differential hypoxia or two-cycle syndrome, results from a high afterload (physiological obstruction) and recovered left ventricular systolic function [69]. Currently, the clinical detection of upper body hypoxia is performed by monitoring saturation in the right radial artery, which reflects the patient’s cardiac output. In addition, near-infrared reflectance spectroscopy (NIRS) can be used to monitor tissue oxygen saturation. Upper body hypoxia is suspected when the regional oxygen saturation (rSO_2_) drops below 40 or decreases more than 25% from baseline or a delta-rSO_2_ between the right radial artery and left radial artery > 15% is detected by NIRS [70]. Approaches to alleviating hypoxia in the upper body include (1) adjusting the ventilator parameters, including increasing the oxygen supplementation and PEEP; (2) increasing the ECMO flow with full drainage of the superior vena cava; (3) choosing the internal jugular vein or femoral vein for venous catheterization, in which case the tip of the catheter is located in the middle of the right atrium [71]; and (4) reducing the risk of limb ischaemia using a distal limb perfusion catheter. V-VA ECMO is used if the condition cannot be alleviated using the previous approaches.

#### V-VA ECMO

The initiation of V-VA ECMO as a pre-emptive strategy is not conventionally recommended. It should only be used in experienced centres for patients with suspected acute stress/septic cardiomyopathy or massive pulmonary embolism or associated cardiogenic/obstructive shock that is not responsive to medical therapies. The occurrence of refractory upper body hypoxia in V-A ECMO is also an indication for establishing V-VA ECMO [72]. In the V-VA ECMO mode, arterial outflow is separated using a Y-connector to deliver well-oxygenated blood to the venous system. The oxygen content output by the left ventricle circulation increases through the pulmonary circulation, which is equivalent to combining V-A ECMO and V-V ECMO in the same circuit. In the application of V-VA ECMO, the flow of these two perfusion circuits should be monitored separately to achieve simultaneous heart and lung support.

## Discussion

ECMO, a type of ECLS, is a breathing and circulation support technology that supplements the functioning of the lungs and heart. It has been widely used in the treatment of various critically ill patients with respiratory and/or circulatory failure in the past decade. However, the provision of therapy with ECMO during the outbreak of an emerging contagious disease is challenging.

In patients with indications for ECMO therapy and without obvious contraindications, ECMO therapy should be initiated in a timely manner. Having a complete understanding of the support provided by different ECMO modes and the differential distribution of oxygen under different ECMO modes, performing a full assessment the patient's heart and respiratory function and selecting the appropriate ECMO mode can improve survival. Several factors, such as sex, age, comorbidities, clinical manifestations and the duration of mechanical ventilation, can affect the outcomes of ECMO [73, 74].

The number of critically ill patients with COVID-19 is increasing rapidly every day, causing a shortage of ECMO devices in some regions. Therefore, it is crucial to prioritize the most eligible patients for ECMO therapy. ECMO should be used preferentially for a certain group of patients (young age, absence of comorbidities, low risk of bleeding, ischaemia, and infectious complications) because they have higher survival rates. Governments should invest in more ECMO devices, ventilators and related medical equipment to support more patients.

## Conclusion

Patients with COVID-19 complicated with severe ARDS have a high mortality rate. The use of ECMO can further complicate the pathophysiological state in COVID-19 patients. However, several studies have reported that the appropriate use of ECMO improves the prognosis of patients. COVID-19 is a major cause of ARDS, which causes most COVID-19-related deaths. This study provides information about the effective use of ECMO in critically ill COVID-19 patients. However, these recommendations should continue to be updated and improved as additional high-quality trials are completed.

## Data Availability

Not applicable.

## Abbreviations

ECMO: Extracorporeal membrane oxygenation
COVID-19: Coronavirus disease 2019
SARS-CoV-2: Severe acute respiratory syndrome coronavirus 2
ARDS: Acute respiratory distress syndrome
WHO: World Health Organization
V-V: Veno-venous
V-A: Veno-arterial
V-VA: Veno-venous arterial
ACE2: Angiotensin-converting enzyme 2
ICU: Intensive care unit
DIC: Disseminated intravascular coagulation
CRP: C-reactive protein
AKI: Acute kidney injury
ECLS: Extracorporeal life support
VILI: Ventilator-induced lung injury
MIS-C: Multisystem inflammatory syndrome in children
PEEP: Positive end-expiratory pressure
NIRS: Near-infrared reflectance spectroscopy
rSO_2_: Regional oxygen saturation

## References

[1] Wu JT, Leung K, Leung GM (2020). Nowcasting and forecasting the potential domestic and international spread of the 2019-nCoV outbreak originating in Wuhan, China: a modelling study. Lancet.

[2] Coronavirus resource center of Johns Hopkins University. https://coronavirus.jhu.edu/map.html. Accessed 22 Jan 2021.

[3] Chen N, Zhou M, Dong X, Qu J, Gong F, Han Y, Qiu Y, Wang J, Liu Y, Wei Y (2020). Epidemiological and clinical characteristics of 99 cases of 2019 novel coronavirus pneumonia in Wuhan, China: a descriptive study. Lancet.

[4] Helms J, Tacquard C, Severac F, Leonard-Lorant I, Ohana M, Delabranche X, Merdji H, Clere-Jehl R, Schenck M, Fagot GF (2020). High risk of thrombosis in patients with severe SARS-CoV-2 infection: a multicenter prospective cohort study. Intensive Care Med.

[5] Cummings MJ, Baldwin MR, Abrams D, Jacobson SD, Meyer BJ, Balough EM, Aaron JG, Claassen J, Rabbani LE, Hastie J (2020). Epidemiology, clinical course, and outcomes of critically ill adults with COVID-19 in New York City: a prospective cohort study. Lancet.

[6] Rieder M, Wengenmayer T, Staudacher D, Duerschmied D, Supady A (2020). Cytokine adsorption in patients with severe COVID-19 pneumonia requiring extracorporeal membrane oxygenation. Crit Care.

[7] Ruan Q, Yang K, Wang W, Jiang L, Song J (2020). Clinical predictors of mortality due to COVID-19 based on an analysis of data of 150 patients from Wuhan, China. Intensive Care Med.

[8] Gattinoni L, Chiumello D, Caironi P, Busana M, Romitti F, Brazzi L, Camporota L (2020). COVID-19 pneumonia: different respiratory treatments for different phenotypes?. Intensive Care Med.

[9] Daviet F, Guervilly C, Baldesi O, Bernard-Guervilly F, Pilarczyk E, Genin A, Lefebvre L, Forel JM, Papazian L, Camoin-Jau L (2020). Heparin-induced thrombocytopenia in severe COVID-19. Circulation.

[10] Yang X, Hu M, Yu Y, Zhang X, Fang M, Lian Y, Peng Y, Wu L, Wu Y, Yi J (2020). Extracorporeal membrane oxygenation for SARS-CoV-2 acute respiratory distress syndrome: a retrospective study from Hubei, China. Front Med (Lausanne).

[11] Schmidt M, Hajage D, Lebreton G, Monsel A, Voiriot G, Levy D, Baron E, Beurton A, Chommeloux J, Meng P (2020). Extracorporeal membrane oxygenation for severe acute respiratory distress syndrome associated with COVID-19: a retrospective cohort study. Lancet Respir Med.

[12] Zeng Y, Cai Z, Xianyu Y, Yang BX, Song T, Yan Q (2020). Prognosis when using extracorporeal membrane oxygenation (ECMO) for critically ill COVID-19 patients in China: a retrospective case series. Crit Care.

[13] Arentz M, Yim E, Klaff L, Lokhandwala S, Riedo FX, Chong M, Lee M (2020). Characteristics and outcomes of 21 critically ill patients with COVID-19 in Washington State. JAMA.

[14] Grasselli G, Zangrillo A, Zanella A, Antonelli M, Cabrini L, Castelli A, Cereda D, Coluccello A, Foti G, Fumagalli R (2020). Baseline characteristics and outcomes of 1591 patients infected with SARS-CoV-2 admitted to ICUs of the Lombardy Region, Italy. JAMA.

[15] Yang X, Yu Y, Xu J, Shu H, Xia J, Liu H, Wu Y, Zhang L, Yu Z, Fang M (2020). Clinical course and outcomes of critically ill patients with SARS-CoV-2 pneumonia in Wuhan, China: a single-centered, retrospective, observational study. Lancet Respir Med.

[16] Barbaro RP, MacLaren G, Boonstra PS, Iwashyna TJ, Slutsky AS, Fan E, Bartlett RH, Tonna JE, Hyslop R, Fanning JJ (2020). Extracorporeal membrane oxygenation support in COVID-19: an international cohort study of the Extracorporeal Life Support Organization registry. Lancet.

[17] World Health Organization. Clinical management of severe acute respiratory infection when novel coronavirus (2019-nCoV) infection is suspected-interim guidance. https://www.who.int/publications-detail/clinical-management-of-severe-acute-respiratory-infection-when-novel-coronavirus-(ncov)-infection-is-suspected. Accessed 17 Aug 2020.

[18] Chinese Society of Extracorporeal Life Support (2020). Recommendations on extracorporeal life support for critically ill patients with novel coronavirus pneumonia. Chin J Tuberc Respir Dis.

[19] MacLaren G, Fisher D, Brodie D (2020). Preparing for the most critically ill patients with COVID-19: the potential role of extracorporeal membrane oxygenation. JAMA.

[20] Brodie D, Slutsky AS, Combes A (2019). Extracorporeal life support for adults with respiratory failure and related indications: a review. JAMA.

[21] Cho HJ, Heinsar S, Jeong IS, Shekar K, Li BG, Jung JS, Suen JY, Fraser JF (2020). ECMO use in COVID-19: lessons from past respiratory virus outbreaks—a narrative review. Crit Care.

[22] Shekar K, Badulak J, Peek G, Boeken U, Dalton HJ, Arora L, Zakhary B, Ramanathan K, Starr J, Akkanti B (2020). Extracorporeal life support organization coronavirus disease 2019 interim guidelines: a consensus document from an International Group of Interdisciplinary Extracorporeal Membrane Oxygenation Providers. ASAIO J.

[23] Lu R, Zhao X, Li J, Niu P, Yang B, Wu H, Wang W, Song H, Huang B, Zhu N (2020). Genomic characterisation and epidemiology of 2019 novel coronavirus: implications for virus origins and receptor binding. Lancet.

[24] Ziehr DR, Alladina J, Petri CR, Maley JH, Moskowitz A, Medoff BD, Hibbert KA, Thompson BT, Hardin CC (2020). Respiratory pathophysiology of mechanically ventilated patients with COVID-19: a cohort study. Am J Respir Crit Care Med.

[25] Osho AA, Moonsamy P, Hibbert KA, Shelton KT, Trahanas JM, Attia RQ, Bloom JP, Onwugbufor MT, D'Alessandro DA, Villavicencio MA (2020). Veno-venous extracorporeal membrane oxygenation for respiratory failure in COVID-19 patients: early experience from a major academic medical center in North America. Ann Surg.

[26] Zhou F, Yu T, Du R, Fan G, Liu Y, Liu Z, Xiang J, Wang Y, Song B, Gu X (2020). Clinical course and risk factors for mortality of adult inpatients with COVID-19 in Wuhan, China: a retrospective cohort study. Lancet.

[27] Xu X, Yu C, Qu J, Zhang L, Jiang S, Huang D, Chen B, Zhang Z, Guan W, Ling Z (2020). Imaging and clinical features of patients with 2019 novel coronavirus SARS-CoV-2. Eur J Nucl Med Mol Imaging.

[28] Chung M, Bernheim A, Mei X, Zhang N, Huang M, Zeng X, Cui J, Xu W, Yang Y, Fayad ZA (2020). CT imaging features of 2019 Novel Coronavirus (2019-nCoV). Radiology.

[29] Mak SM, Mak D, Hodson D, Preston R, Retter A, Camporota L, Benedetti G (2020). Pulmonary ischaemia without pulmonary arterial thrombus in COVID-19 patients receiving extracorporeal membrane oxygenation: a cohort study. Clin Radiol.

[30] Wu C, Chen X, Cai Y, Xia J, Zhou X, Xu S, Huang H, Zhang L, Zhou X, Du C (2020). Risk factors associated with acute respiratory distress syndrome and death in patients with coronavirus disease 2019 pneumonia in Wuhan, China. JAMA Intern Med.

[31] Adamos G, Gavrielatou E, Sarri K, Kokkoris S (2020). Heterogeneity of acute respiratory distress syndrome. Am J Respir Crit Care Med.

[32] Guo T, Fan Y, Chen M, Wu X, Zhang L, He T, Wang H, Wan J, Wang X, Lu Z (2020). Cardiovascular implications of fatal outcomes of patients with coronavirus disease 2019 (COVID-19). JAMA Cardiol.

[33] Lippi G, Lavie CJ, Sanchis-Gomar F (2020). Cardiac troponin I in patients with coronavirus disease 2019 (COVID-19): evidence from a meta-analysis. Prog Cardiovasc Dis.

[34] Bangalore S, Sharma A, Slotwiner A, Yatskar L, Harari R, Shah B, Ibrahim H, Friedman GH, Thompson C, Alviar CL (2020). ST-segment elevation in patients with Covid-19—a case series. N Engl J Med.

[35] Sala S, Peretto G, Gramegna M, Palmisano A, Villatore A, Vignale D, De Cobelli F, Tresoldi M, Cappelletti AM, Basso C (2020). Acute myocarditis presenting as a reverse Tako-Tsubo syndrome in a patient with SARS-CoV-2 respiratory infection. Eur Heart J.

[36] Tavazzi G, Pellegrini C, Maurelli M, Belliato M, Sciutti F, Bottazzi A, Sepe PA, Resasco T, Camporotondo R, Bruno R (2020). Myocardial localization of coronavirus in COVID-19 cardiogenic shock. Eur J Heart Fail.

[37] Long B, Brady WJ, Koyfman A, Gottlieb M (2020). Cardiovascular complications in COVID-19. Am J Emerg Med.

[38] Dong N, Cai J, Zhou Y, Liu J, Li F (2020). End-stage heart failure with COVID-19: strong evidence of myocardial injury by 2019-nCoV. JACC Heart Fail.

[39] Klok FA, Kruip M, van der Meer N, Arbous MS, Gommers D, Kant KM, Kaptein F, van Paassen J, Stals M, Huisman MV (2020). Incidence of thrombotic complications in critically ill ICU patients with COVID-19. Thromb Res.

[40] Mazzoni A, Salvati L, Maggi L, Capone M, Vanni A, Spinicci M, Mencarini J, Caporale R, Peruzzi B, Antonelli A (2020). Impaired immune cell cytotoxicity in severe COVID-19 is IL-6 dependent. J Clin Invest.

[41] Henry BM, de Oliveira M, Benoit S, Plebani M, Lippi G (2020). Hematologic, biochemical and immune biomarker abnormalities associated with severe illness and mortality in coronavirus disease 2019 (COVID-19): a meta-analysis. Clin Chem Lab Med.

[42] Rothstein A, Oldridge O, Schwennesen H, Do D, Cucchiara BL (2020). Acute cerebrovascular events in hospitalized COVID-19 patients. Stroke.

[43] Morassi M, Bagatto D, Cobelli M, D'Agostini S, Gigli GL, Bna C, Vogrig A (2020). Stroke in patients with SARS-CoV-2 infection: case series. J Neurol.

[44] Zhang Y, Xiao M, Zhang S, Xia P, Cao W, Jiang W, Chen H, Ding X, Zhao H, Zhang H (2020). Coagulopathy and antiphospholipid antibodies in patients with Covid-19. N Engl J Med.

[45] Cheng Y, Luo R, Wang K, Zhang M, Wang Z, Dong L, Li J, Yao Y, Ge S, Xu G (2020). Kidney disease is associated with in-hospital death of patients with COVID-19. Kidney Int.

[46] Ronco C, Reis T (2020). Kidney involvement in COVID-19 and rationale for extracorporeal therapies. Nat Rev Nephrol.

[47] Chen T, Wu D, Chen H, Yan W, Yang D, Chen G, Ma K, Xu D, Yu H, Wang H (2020). Clinical characteristics of 113 deceased patients with coronavirus disease 2019: retrospective study. BMJ.

[48] Matthay MA, Aldrich JM, Gotts JE (2020). Treatment for severe acute respiratory distress syndrome from COVID-19. Lancet Respir Med.

[49] Beyls C, Huette P, Abou-Arab O, Berna P, Mahjoub Y (2020). Extracorporeal membrane oxygenation for COVID-19-associated severe acute respiratory distress syndrome and risk of thrombosis. Br J Anaesth.

[50] Li X, Guo Z, Huang J (2020). One disaster after another or a timely help? The role of ECMO for COVID-19 patients. ASAIO J.

[51] Thiagarajan RR, Barbaro RP, Rycus PT, Mcmullan DM, Conrad SA, Fortenberry JD, Paden ML (2017). Extracorporeal Life Support Organization Registry International Report 2016. ASAIO J.

[52] Pham DT, Toeg H, De Paulis R, Atluri P (2020). Establishment and management of mechanical circulatory support during the COVID-19 pandemic. Circulation.

[53] Belhadjer Z, Meot M, Bajolle F, Khraiche D, Legendre A, Abakka S, Auriau J, Grimaud M, Oualha M, Beghetti M (2020). 25 Syndrome in children in the context of global SARS-CoV-2 pandemic. Circulation.

[54] Jacobs JP, Stammers AH, St LJ, Hayanga J, Firstenberg MS, Mongero LB, Tesdahl EA, Rajagopal K, Cheema FH, Coley T (2020). Extracorporeal membrane oxygenation in the treatment of severe pulmonary and cardiac compromise in coronavirus disease 2019: experience with 32 patients. ASAIO J.

[55] Le Breton C, Besset S, Freita-Ramos S, Amouretti M, Billiet PA, Dao M, Dumont LM, Federici L, Gaborieau B, Longrois D (2020). Extracorporeal membrane oxygenation for refractory COVID-19 acute respiratory distress syndrome. J Crit CARE.

[56] Zhang G, Hu C, Luo L, Fang F, Chen Y, Li J, Peng Z, Pan H (2020). Clinical features and short-term outcomes of 221 patients with COVID-19 in Wuhan, China. J Clin Virol.

[57] Li X, Guo Z, Li B, Zhang X, Tian R, Wu W, Zhang Z, Lu Y, Chen N, Clifford SP (2020). Extracorporeal membrane oxygenation for coronavirus disease 2019 in Shanghai, China. ASAIO J.

[58] Kon ZN, Smith DE, Chang SH, Goldenberg RM, Angel LF, Carillo JA, Geraci TC, Cerfolio RJ, Montgomery RA, Moazami N (2021). Extracorporeal membrane oxygenation support in severe COVID-19. Ann Thorac Surg.

[59] Schmidt M, Pham T, Arcadipane A, Agerstrand C, Ohshimo S, Pellegrino V, Vuylsteke A, Guervilly C, McGuinness S, Pierard S (2019). Mechanical ventilation management during extracorporeal membrane oxygenation for acute respiratory distress syndrome. An international multicenter prospective cohort. Am J Respir Crit Care Med.

[60] Tang X, Wu C, Li X, Song Y, Lu J (2020). On the origin and continuing evolution of SARS-CoV-2. Natl Sci Rev.

[61] Wang X, Ma S. Demand for medical supplies in Hubei ‘have been satisfied’. http://www.gov.cn/statecouncil/ministries/www.gov.cn/statecouncil/ministries/202003/05/content_WS5e605862c6d0c201c2cbd9ac.html. Accessed 17 Aug 2020

[62] Dechert RE, Park PK, Bartlett RH (2014). Evaluation of the oxygenation index in adult respiratory failure. J Trauma Acute Care Surg.

[63] Villar J, Ambros A, Soler JA, Martinez D, Ferrando C, Solano R, Mosteiro F, Blanco J, Martin-Rodriguez C, Fernandez MM (2016). Age, PaO_2_/FIO_2_, and plateau pressure score: a proposal for a simple outcome score in patients with the acute respiratory distress syndrome. Crit Care Med.

[64] Shaefi S, Brenner SK, Gupta S, O'Gara BP, Krajewski ML, Charytan DM, Chaudhry S, Mirza SH, Peev V, Anderson M (2021). Extracorporeal membrane oxygenation in patients with severe respiratory failure from COVID-19. Intensive Care Med.

[65] MacLaren G, Combes A, Bartlett RH (2012). Contemporary extracorporeal membrane oxygenation for adult respiratory failure: life support in the new era. Intensive Care Med.

[66] Doufle G, Roscoe A, Billia F, Fan E (2015). Echocardiography for adult patients supported with extracorporeal membrane oxygenation. Crit Care.

[67] Hoeper MM, Wiesner O, Hadem J, Wahl O, Suhling H, Duesberg C, Sommer W, Warnecke G, Greer M, Boenisch O (2013). Extracorporeal membrane oxygenation instead of invasive mechanical ventilation in patients with acute respiratory distress syndrome. Intensive Care Med.

[68] Burrell A, Ihle JF, Pellegrino VA, Sheldrake J, Nixon PT (2018). Cannulation technique: femoro-femoral. J Thorac Dis.

[69] Cove ME (2015). Disrupting differential hypoxia in peripheral veno-arterial extracorporeal membrane oxygenation. Crit Care.

[70] Wong JK, Smith TN, Pitcher HT, Hirose H, Cavarocchi NC (2012). Cerebral and lower limb near-infrared spectroscopy in adults on extracorporeal membrane oxygenation. Artif Organs.

[71] Hou X, Yang X, Du Z, Xing J, Li H, Jiang C, Wang J, Xing Z, Li S, Li X (2015). Superior vena cava drainage improves upper body oxygenation during veno-arterial extracorporeal membrane oxygenation in sheep. Crit Care.

[72] Bonicolini E, Martucci G, Simons J, Raffa GM, Spina C, Coco VL, Arcadipane A, Pilato M, Lorusso R (2019). Limb ischemia in peripheral veno-arterial extracorporeal membrane oxygenation: a narrative review of incidence, prevention, monitoring, and treatment. Crit Care.

[73] Brechot N, Mastroianni C, Schmidt M, Santi F, Lebreton G, Hoareau AM, Luyt CE, Chommeloux J, Rigolet M, Lebbah S (2018). Retrieval of severe acute respiratory failure patients on extracorporeal membrane oxygenation: any impact on their outcomes?. J Thorac Cardiovasc Surg.

[74] Serpa NA, Schmidt M, Azevedo LC, Bein T, Brochard L, Beutel G, Combes A, Costa EL, Hodgson C, Lindskov C (2016). Associations between ventilator settings during extracorporeal membrane oxygenation for refractory hypoxemia and outcome in patients with acute respiratory distress syndrome: a pooled individual patient data analysis: mechanical ventilation during ECMO. Intensive Care Med.

